# Effects of Poultry By-Product Composition and Processing on Nutrient Digestibility and Fecal Characteristics of High-Protein Dry Dog Food

**DOI:** 10.3390/ani15182693

**Published:** 2025-09-15

**Authors:** Lisa Brands, Cristina Ullrich, Volker Wilke, Christian Visscher, Josef Kamphues, Amr Abd El-Wahab

**Affiliations:** 1 Institute for Animal Nutrition, University of Veterinary Medicine Hannover, Foundation, Bischofsholer Damm 15, D-30173 Hannover, Germanycristina.ullrich@tiho-hannover.de (C.U.); volker.wilke@tiho-hannover.de (V.W.); christian.visscher@tiho-hannover.de (C.V.); josef.kamphues.ir@tiho-hannover.de (J.K.); 2 Department of Nutrition and Clinical Nutrition, Faculty of Veterinary Medicine, Mansoura University, Mansoura 35516, Egypt

**Keywords:** feather meal, processing, dogs, digestibility, sustainability

## Abstract

Poultry by-products, particularly feather meal (FM), are discussed as promising sustainable protein sources for pet foods. This study aimed to evaluate the impact of FMs, differing in processing and origin, when incorporated into a complete diet across two studies on nutrient digestibility and fecal quality in dogs. Six dogs participated in a crossover experimental design. Firstly, premium FM (FMpre), dried at 270 °C for 60 s, followed by drying at 80 °C for 120 s, and economy FM (FMeco I), dried at 160 °C for 60 min, were provided. FMpre contained a mixture of poultry feathers, FMeco I consisted of broiler feathers. Afterwards, FMpre was compared to FMeco II, which also consisted of a poultry feather mix, except it was one processed under the same conditions as FMeco I. In the first trial, no significant differences in apparent nutrient digestibility were observed between the two diets. The second trial, however, showed a significantly lower apparent organic matter digestibility of FMeco II compared to FMpre. Generally, no significant differences in fecal quality scores were observed. Moreover, the origin of the feathers did not significantly affect the nutritional digestibility or fecal characteristics of the dogs.

## Simple Summary

Slaughter by-products such as feather meal are rendered (heat-processed) to produce sustainable, high-quality protein ingredients for dog food. The processing differentiates between premium feather meals (dried at 270 °C for 60 s, followed by drying at 80 °C for 120 s) and economy feather meals (dried at 160 °C for 60 min). This study aimed to evaluate the potential impacts of these qualitative differences on the digestibility and fecal quality of six dogs across two trials. The feather meals, varying in processing and composition, were incorporated into a complete diet and used for feeding for seven days: the premium diet included feathers from broilers, turkeys, and laying hens, as did the economy II diet, whereas the economy I diet only contained broiler feathers. In the first trial (premium vs. economy I), there were no significant differences in nutrient digestibility. In the second trial (premium vs. economy II), dogs fed the economy II diet showed significantly lower digestibility of organic matter compared to the premium product. Additionally, the source of the feathers did not significantly impact the dogs’ nutrient digestibility or fecal traits.

## 1. Introduction

The demand for protein sources has risen in recent years and is expected to increase significantly in the future, potentially intensifying competition between humans and animals for these sources [1]. Therefore, the utilization of all available protein sources, including slaughter by-products such as feather meal (FM), is essential to reduce competition for high-quality proteins and to support sustainability.

In general, the global market for protein-based ingredients was estimated at USD 38 billion in 2019 and is expected to grow at a compound annual growth rate of 9.1% until 2027 [2]. Global population growth is projected to reach 9.7 billion by 2050 [3]. In addition, the global production of poultry meat rose by 150 million tons in 2024, marking a 2.6 percent increase compared with 2023 [4]. Consequently, the global demand for animal-derived protein is anticipated to double by 2050, raising concerns about sustainability and food security [5]. Moreover, Raspa et al. [6] underline the need to formulate specific practical guidelines for safe feeding and bowl hygiene practices to minimize the risk of microbiological contamination in the domestic environment. In addition, the number of pet owners continues to increase worldwide. Taking into account households with several animals, it is estimated that there were 104 million pet dogs in Europe alone in 2022, an increase of 29% compared to a total of 74 million dogs in 2010 [7]. These figures further underline the importance of research into viable and sustainable protein sources for pets, including slaughter by-products.

Animal by-products, which are the main ingredient responsible for the growth of the global pet food industry, provide the most dietary protein [8,9]. In the European Union, over 20 million metric tons of animal by-products are produced annually from slaughterhouses and food-processing industries, as well as from on-farm mortalities, which are categorized as high-risk materials and excluded from feed use under EU law [10]. Feathers, a by-product of poultry slaughterhouses, account for about 7% of the bird’s body weight [11]. According to the Food and Agriculture Organization (FAO) [12], global poultry meat output in 2023 was projected to reach 142 million tonnes, representing a 1% year-on-year increase. Much of the expected increase in production was concentrated in China, Brazil, and the European Union. In terms of international trade, global poultry meat exports were forecast to reach 16.3 million tonnes in 2023, a modest 0.7% increase from 2022 levels. This growth was expected to be driven by rising imports in Asia, Central America and the Caribbean, and Europe, partially offset by declines in other regions. 

FM typically contains over 85% crude protein (CP) in dry matter (DM), primarily from keratin, the main constituent of feathers [13]. Feathers are converted to hydrolyzed FM in the industry by breaking them down through thermal processing at high pressure and high temperature, resulting in a digestible ingredient with a high protein concentration. Hydrolyzed FM offers an inexpensive protein source for inclusion in dog and cat diets [14]. Despite the focus of the present study, hydrolyzed FM has limited practical use in companion animal diets and is generally included only at low levels in formulations, mainly due to its amino acid profile and palatability constraints [13,15]. The high temperatures and pressure during processing are necessary to disrupt the keratin structure in the feathers, thus making the proteins digestible and absorbable for animals [13]. However, prolonged exposure of feathers to these conditions can reduce the availability of amino acids, particularly cysteine, which is sensitive to temperature [16,17]. Despite the recognized potential of FM as an alternative protein source, information is still limited regarding how production processes (e.g., drying methods, hydrolysis systems) and the poultry species of origin influence its nutritional quality and digestibility in companion animal diets.

There is a growing interest in alternative protein sources to meet market as well as consumer demand and to enhance sustainability by utilizing by-products from other industries. To support a sustainable future, this vast amount of material must be managed using safe, environmentally responsible and efficient methods to recover valuable resources [18]. Against this background, the objective of this study was to evaluate the effects of different FM treatments/processes and the origin of feathers in Beagle dog diets on apparent nutrient digestibility and fecal quality (including fecal consistency and form scores).

## 2. Materials and Methods

Prior to conducting this study, the study protocol was evaluated and approved by the Animal Welfare Officer of the University of Veterinary Medicine Hannover, Foundation, Hannover (TiHo), Germany in accordance with the German protocol § 7 of the Animal Protection Law (approval number TVG-2017-V-66/2017-04/20).

### 2.1. Experimental Design and Dietary Treatments

Six intact female Beagle dogs participated in this study, which consisted of two trials. All dogs originated from the TiHo. At the beginning of the study, the dogs had a mean body weight (BW) of 9.73 ± 1.08 kg and ranged in age from 2 to 5 years. Body condition scoring (9-point scoring system, values of 4 or 5 being considered ideal) was performed by the same investigator at the beginning and end of each of the feeding trials according to Laflamme [19]. The median body condition score was 4.98 of 9 over the entire experimental trial. The study was executed using a crossover experimental design with two experimental diets in each segment. Animals were divided into two groups of three dogs each. For each trial, the dogs underwent a 5-day acclimation period [7], followed by 5 days of fecal collection for individual estimation of apparent nutrient digestibility and fecal scores. Five days served as a washout period before initiating both experimental periods, which included adaptation and collection phases.

In accordance with the manufacturer, the FM used in both studies derived from different processing methods and origin. Hydrolyzed feather meal samples (FMeco I, FMeco II, FMpre) were obtained through a German commercial supplier. Detailed company information is not available; however, the samples represent commercially produced feather meals commonly used in the feed industry.

### 2.2. Food Processing

#### 2.2.1. First Trial

A high-quality FM, hereinafter referred to as premium FM (FMpre), and a low-quality FM, hereinafter referred to as economy FM (FMeco I), were used for comparison. The manufacturer’s classification into different quality grades is associated with differences in the technological preparation of the FM and their various origins. While the raw materials of both FM types initially underwent an identical hydrolysis, they were subjected to different drying processes thereafter.

The manufacturer described the processing techniques of the feather meals used in this phase as follows: First, the raw materials of both feather meals were heated to approximately 100 °C to break the initial disulfide bonds in the keratin structure of the feathers and initiate hydrolysis. Processing at about 143 °C and a pressure of 4 bar subsequently produced “feather greaves.” Since the resulting material at this stage had a moisture content of around 70%, two different drying procedures followed after pressing out the excess water: FMeco I was dried for 60 min at 160 °C in a disk dryer, whereas the processing of FMpre involved a two-step drying procedure. In a pre-dryer, the material was first dried for about 60 s at 270 °C, followed by drying in a post-dryer at 80 °C for 120 s. Unlike the processing of FMeco I, the drying of FMpre took place in a stream of hot air without direct contact between the material and heated surfaces.

Another difference was the origin and composition of the FM. While the FMeco I consisted of 100% broiler feathers, the FMpre contained feathers from turkeys, broilers, and laying hens. Both FMs were included at 25% in two complete dog foods of identical composition. An overview of the composition of these two diets is given in Table 1.

#### 2.2.2. Second Trial

In the second trial, FMpre was identical to that used in the first trial (same composition and processing). Similarly, FMeco II was produced from feathers of turkeys, broilers, and laying hens using the same processing conditions as FMeco I. The FMs (FMpre and FMeco II) were included at 25% and 25.8% in two complete diets, respectively, as shown in Table 2.

### 2.3. Food Intake and Scoring

The required amount of feed for adequate energy supply, particularly for maintaining constant body weight across the respective trial, was determined in the first experiment using the following formula to calculate energy needs: 0.40 MJ of metabolizable energy × BW^0.75^/day, and in the second trial: 0.45 MJ of metabolizable energy × BW^0.75^/day. Food allowance was based on the maintenance needs of the heaviest dog, with BW determined at the beginning and end of each trial and supported by body condition scoring. An energy requirement of 0.40 MJ ME × BW^0.75^/day was assumed in first trial, while in second trial, which was conducted at a later time point under different seasonal conditions, the requirement was increased to 0.45 MJ ME × BW^0.75^/day to account for potential environmental influences. To predict a mean of the quantity of food offered, we have first to estimate the metabolizable energy contents of the diets based on their chemical composition in accordance with Kamphues et al. [20] according to the following equation: metabolizable energy (MJ/kg) = 0.01674 × crude protein + 0.03767 × crude fat + 0.1674 × nitrogen-free extract. The dogs were fed once per day (at 07:00) and received fresh water ad libitum. Feed intake was determined by measuring the residual feed, calculated as the amount of feed offered minus the remaining amount of feed. In the first trial, the dogs were fed an average of 146 g (FMpre as fed; 134 g DM) and 145 g per day (FMeco I as fed; 133 g DM). In the second trial, the dogs were fed an average of 164 g (FMpre as fed; 151 g DM) and 165 g per day (FMeco II as fed; 156 g DM). Food intake was assessed by offering a pre-weighed portion of the test diet and recording the amount consumed (offered minus residual food). Palatability was further evaluated using a three-point scoring system adapted from Zahn [21], which considers both preference and speed of consumption: score 1 = lowest preference (slow or incomplete consumption), score 2 = moderate preference (steady but not immediate consumption), and score 3 = highest preference (rapid and complete consumption).

### 2.4. Apparent Nutrient Digestibility

The total fecal collection method was used to evaluate the apparent total tract digestibility (ATTD), involving an initial phase of 5 days of adaptation to the diet, followed by 5 days of fecal collection (((food−feces)/food) × 10) [7]. During the collection period, fresh feces were collected daily from the concrete floor. For the five consecutive days of the collecting phase, fecal shedding was checked every 30 min (from 07:00 to 21:00). After being weighed, a subsample of 10% of the fresh feces per animal per day was taken to determine the DM content. Thereafter, the remaining fecal samples were stored at −20 °C. At the end of each trial, the collected fecal samples from each dog, spanning five days, were thawed, mixed, and homogenized to estimate the ATTD of organic matter, CP, crude fat, and nitrogen-free extracts with reference to the initially determined DM.

### 2.5. Fecal Consistency and Form Scoring

The number of defecations per dog and day was recorded. In accordance with Moxham [22], the fecal consistency scores were assessed by the same person and recorded for each defecation using a five-point scale (1 = very hard; 2 = solid, well formed; 3 = soft, still formed; 4 = pasty, slushy; 5 = watery diarrhea). A graphic representation of the fecal scoring system was previously described by Abd El-Wahab et al. [23]. A score of 2, representing solid, well-formed feces, was considered “optimal” [24]. To provide additional details on fecal morphology, such as constrictions and fissures on the stool surface, another scoring system (fecal form) was used in accordance with Zeiger [22], which included four grades (1 = individual fecal pieces, 2 = shaped, with strong constrictions on the surface of the feces, 3 = shaped, with fissures on the surface of the feces, 4 = shapeless).

### 2.6. Chemical Analyses and Diet Composition

To determine nutrients in the diets and fecal samples, the procedures of the Association of German Agricultural Analytical and Research Institutes e.V. were used [25].

### 2.7. Statistical Analysis

The statistical analysis was performed using the Statistical Analysis System for Windows, SAS1 Enterprise Guide1, version 9.3 (SAS Institute, Inc., Cary, NC, USA). For all parameters, mean values along with the standard deviation (SD) of the mean were calculated. For values in the form of a score, such as the fecal consistency score, median values and interquartile range (IQR) were determined. All measured or recorded parameters were analyzed individually and formed the basis of the calculation. Depending on the distribution analysis of the data, both parametric and non-parametric methods were applied. To compare the mean values of the apparent nutrient digestibility and fecal DM content, the normal distribution of the residuals was first tested using a Shapiro–Wilk test [26]. Differences among treatments were determined using a multi-range test (Ryan-Einot-Gabriel-Welsch test). For non-normally distributed data, such as fecal defecation frequency or values in the forming score, the Kruskal–Wallis test was used [27]. For values in the form of a fecal consistency score, two-dimensional frequency distributions of categorical features were checked for dependency using Pearson’s chi-square test of homogeneity, provided the sample was evenly distributed. Otherwise, Fisher’s exact test was used. The significance level was determined at *p* < 0.05.

## 3. Results

### 3.1. Composition of the Feather Meals (FMs)

While the protein content of the two different FMs was comparable in both trials, the crude ash content as well as the content of all major elements in FMpre were nummericaly higher than FMeco I and FMeco II (Table 3).

Table 4 shows the amino acid content (g/kg DM and g/100 CP) of the FMs. Serine, glutamine, and proline contents were numerically highest in FMeco I, whereas the levels of methionine and histidine were numerically the lowest in this diet.

### 3.2. Composition of the Complete Diets

The chemical composition of the complete diets in the first trial was comparable for most nutrients, except for the parameter starch. In the second trial, the crude fat content was numerically higher in the FMeco II diet (Table 5), however, without affecting the energy content, which remained comparable to that of the FMpre diet as well as the protein levels (Table 5).

In Table 6, the amino acid contents (g/kg DM and g/100 CP) of the complete diets are shown. In the first trial, the contents of serine, glutamine, and cysteine were numerically higher in the FMeco I diet. However, in the second trial, the FMeco II diet showed numerically higher contents in most of the amino acids, such as lysine, leucine, and valine.

### 3.3. Nutrient Digestibility of the Complete Diets

In the first trial, there were no significant differences in ATTD between both diets offered to the dogs. Nonetheless, in the second trial, ATTD for organic matter was significantly lower for dogs fed the diet containing FMeco II compared to those fed the FMpre diet (Table 7). Moreover, crude fat digestibility was numerically higher for the diet with FMpre during the second trial, although this difference was not statistically significant.

### 3.4. Fecal Characteristics

Fecal quality data are presented in Table 8. In the first trial, no significant differences were observed in fecal consistency (almost identical scores of about 3.2), fecal form scores, amount of fecal output, or DM content. In the second trial, there were no significant differences between the two groups in either fecal consistency or fecal form scores. However, dogs fed the diet with FMeco II had higher fecal output (42.9 g DM/day) than those fed the diet containing FMpre (39.8 g DM/day). In the second trial, too, there were no significant differences in fecal DM content (30%) between the two groups.

## 4. Discussion

In this study, hydrolyzed FM was included in dry dog food at 25% of the diet, resulting in final diets containing 37–40 g CP/kg diet DM. While the main purpose was to evaluate the general feasibility of using FMs in canine diets, different FMs with varying processing methods were tested. Differences in chemical composition among the FMs were observed, which influenced the nutritional quality of the resulting diets. Differences in crude fat, ash, and amino acid content among the diets were observed and are likely due to both the type of raw material and the processing methods applied. Notably, FMpre showed numerically higher levels of calcium and phosphorus compared to FMeco I and FMeco II. This may reflect the unintended inclusion of non-feather body parts during the feather collection process, such as heads, which can contribute to an elevated crude ash content, particularly in minerals like calcium and phosphorus [28]. Additionally, the origin of the feathers (from broilers, turkeys, or layers) appears to influence nutrient composition. For instance, broilers are typically slaughtered at a younger age (around 35 days), while turkeys are slaughtered much later (approximately 154 days). Such age differences affect feather composition, including protein content, with older birds exhibiting higher crude protein levels [20,29]. This may help explain the observed variation in crude protein among the three FMs. The quality of FM is determined not only by crude protein concentration but also by processing conditions and nutrient availability. High-quality FM is sufficiently but not excessively hydrolyzed, yielding high and consistent digestibility (pepsin digestibility >75–80%), preservation of amino acids-particularly bioavailable lysine-high CP content (80–90% DM), low ash and moisture content, and a homogeneous appearance with few intact feather shafts. It should also be microbiologically safe and of a color indicative of proper processing (neither burnt nor excessively pale) [30]. Low-quality FM, in contrast, may result from inadequate hydrolysis (low digestibility, visible shafts) or excessive heat treatment (loss of reactive lysine, dark color, poor palatability). It may also exhibit high ash (>8%), high moisture (>10%), or batch-to-batch variability, underscoring that CP percentage alone is not an adequate indicator of quality of FM used as a feedstuff for pigs and poultry [30].

In the second feeding trial, the lower crude fat content in the FMeco II diet (90.3 g/kg DM) compared to the FMpre diet (120 g/kg DM) can be attributed to the differing levels of added poultry fat (3.32% vs. 6.38%). Interestingly, despite similar total sulfur contents, a reduced cysteine level (9.47 g/kg DM) was found in the diet with 25% FMpre, resembling the cysteine level in another diet from the first trial. Since the diets did not differ in formulation and all three FMs showed comparable cysteine contents, this discrepancy may be due to analytical variation or other technical factors influencing amino acid determination. Overall, the study highlights how differences in FM source material and processing can impact key nutritional parameters, which should be carefully considered when formulating diets for companion animals. Importantly, all diets exceeded the minimum recommended amino acid levels for adult dogs as outlined by the National Research Council (NRC) [31] and the European Pet Food Industry Federation (FEDIAF) [7], ensuring that the observed variations did not compromise the nutritional adequacy of the diets.

When evaluating the ATTD of nutrients in the present study, only partial support for the assumed benefits of dried FM was found. In the second trial, the diet containing FM (FMpre, dried at 270 °C for 60 s, followed by drying at 80 °C for 120 s) showed significantly higher digestibility of organic matter (*p* = 0.027) compared to the diet with FMeco II. The digestibility of crude fat was numerically higher in the FMpre group. In contrast, the ATTD values for crude protein, nitrogen-free extracts, and starch were comparable across the diets and trials. These findings partially align with an earlier study by Abd El-Wahab et al. [9], which demonstrated no significant differences for DM and apparent crude protein digestibility among treatments (control and FM up to 20%), while dogs fed the control diet had the highest organic matter digestibility (87.2%) compared to other treatments with FM up to 20% (85.5%). The crude fat digestibility was significantly the lowest (95.0%) for dogs fed food containing FM up to 20% in comparison to other treatments (control, FM inclusion of 5 and 10%) [9].

One possible explanation for the lower organic matter digestibility observed with the FMeco II diet could be its higher crude fiber content (30.7 g/kg DM) compared to FMpre (25.7 g/kg DM). Elevated fiber levels can negatively influence digestibility due to the low digestibility of fibrous materials as well as increasing intestinal viscosity and accelerating chyme passage [32,33]. Similar reductions in ATTD of organic matter (80.0% vs. 83.4%) after using high-fiber diets (46.9 g/kg DM vs. 24.5 g/kg DM) in dogs were also noted in a previous study by Abd El-Wahab et al. [34]. In the present study, organic matter digestibility averaged about 75%, with dry matter digestibility in the low 70 s, indicating relatively modest digestibility for an extruded dog diet. This likely reflects the keratin-rich composition of FM, higher dietary ash, and processing effects that reduce amino acid availability. Practically, these values are below typical commercial targets, so FM inclusion should be limited and balanced with highly digestible protein and energy sources to improve ATTD.

Despite the differences in drying methods between FMpre and FMeco II, no advantage was observed for FMpre in terms of crude protein digestibility. This may be due to the extrusion process during diet preparation, which exposes all ingredients to high temperatures, moisture, and shear forces, potentially reducing the effect of initial protein quality [16].

When evaluating the fecal mass produced over the five-day collection period, a significant difference (*p* = 0.004) was observed in the second trial. This difference can be explained by the 3.31% higher daily dry matter intake (151 g DM vs. 156 g DM) in the diet containing 25% FMeco II, combined with its lower ATTD of organic matter (77.3% vs. 75.6%). The lower digestibility observed for FMeco II may be related not only to its higher fiber content but also to processing-related effects that can decrease nutrient availability. For example, changes in protein structure during rendering or drying, the formation of Maillard reaction products, or reduced amino acid availability may have contributed to less efficient nutrient and energy utilization, thereby increasing fecal mass output. On the other hand, with the FMeco II diet, a significantly (*p* = 0.027) lower ATTD of organic matter (77.3% vs. 75.6%) was measured, which causes a higher output of fecal mass. The effect of causing a higher fecal mass is congruent with results obtained when 15% FM was fed to dogs in a study conducted by Pacheco et al. [13]. Although fecal consistency after using all three diet variants showed a notable deviation from the optimal level (score 2), the results were largely consistent. The fecal consistency scores for the diet containing 25% FMpre and the diet containing 25% FMeco II were 3.23 and 3.24, respectively, and 3.19 (FMpre diet) and 3.05 (FMeco II diet) for the diets in the second trial. Moreover, the values for the fecal form score were comparable for all diets in both trials.

Generally unfavorable effects on fecal quality could be attributed to factors such as the “hydrophobic” characteristics of feather keratins [13,35] and the intensive bacterial conversion of prececal, poorly digestible protein sources [36]. However, no differences were observed between the diets regarding fecal consistency and fecal form scores, showing almost congruent patterns. Considering the high proportion of FM in the diets’ variants (25%), deliberately chosen to clearly highlight the effects on apparent nutrient digestibility and fecal parameters, it is crucial to take this factor into account. These observations align with the findings of Abd El-Wahab et al. [9] who indicated significant variance in fecal consistency between the basal diet and foods supplemented with FM up to 20%, with the latter resulting in significantly softer fecal consistency (score 4 for food containing 20% FM vs. score 2 for control basal food). Research has shown that high proportions of certain animal-derived proteins in diets result in softer feces compared to those with plant-derived protein components [35]. This phenomenon is primarily attributed to the inclusion of low-quality proteins with limited prececal digestibility. Additionally, increased collagen concentrations and the adverse effects of thermal treatment of components contribute to this effect [16]. In a study by Ingenpaß et al. [24], the scores for fecal consistency and shaping were very close to the desired optimum score (score 2) in groups fed meat-based or plant-based diets.

Contrary to expectations, the extra “gentle” drying of FMs had no substantial effect on digestibility, fecal mass, or fecal quality in dogs. Ultimately, the more expensive and technologically complex drying process for FMs did not yield any noticeable benefits for nutrient digestibility and fecal quality in this study. In the context of FM processing, with subsequent incorporation of the extruded product as a protein source in pet food, conventional drying appears to be sufficient in terms of digestibility.

## 5. Conclusions

Over the last years, there has been continued interest in alternative protein sources to satisfy the growing market and consumer demands as well as in using by-products from other industries to improve sustainability. The sustainability of pet keeping is greatly enhanced by recycling animal by-products like FM during the rendering process and using those by-products as feed ingredients for pets. In the present study, dogs responded well to the inclusion of poultry by-products, namely different feather meals, in their diets in terms of nutrient digestibility.

While premium feather meal (FMpre) showed a marginal improvement in organic matter digestibility, it did not significantly enhance overall nutrient absorption or fecal quality compared to the more commonly processed FMeco I and FMeco II. These findings suggest that these three types of feather meal despite their different processing techniques and composition can serve as effective protein sources in dog diets without markedly affecting digestibility or causing notable changes in fecal characteristics. Future research should continue to explore the balance between processing techniques and the nutritional efficacy of feather meals in pet diets to optimize both sustainability and dietary benefits. Although no significant effects of feather meal inclusion were observed in the present study, it should be noted that different production conditions, such as processing technology (e.g., extrusion vs. canning) and inclusion levels, may yield different outcomes. Future research evaluating the digestibility of raw materials or the incorporation of feather meal in canned pet foods would be valuable to clarify these potential differences.

Based on our results, very high inclusion levels of hydrolyzed feather meal reduced protein digestibility in dogs. Further research is needed to determine whether moderate inclusion levels, in combination with balanced dietary protein concentrations, would avoid such negative effects.

## Figures and Tables

**Table 1:** Composition of both experimental complete diets including FMpre and FMeco I.

Item (%)	FMpre *	FMeco I *
Wheat	31.5	31.5
FMpre	25.0	-
FMeco I	-	25.0
Dried distillers grains	16.4	16.4
Wheat bran	10.0	10.0
Meat bone meal	8.38	8.38
Poultry fat	6.38	6.38
Sodium chloride	0.900	0.900
Choline chloride	0.088	0.088
Vitamin E	0.004	0.004
Minerals and vitamins premix ^1^	1.28	1.30

* FMpre: diet with feather meal premium. FMeco I: diet with feather meal economy I. ^1^ The premix used in the diets was a commercial preparation designed to meet the recommended requirements for vitamins and minerals. The detailed ingredient composition of the premix was not available.

**Table 2:** Composition of both experimental complete diets including FMpre and FMeco II.

Item (%)	FMpre *	FMeco II *
Wheat	31.5	32.6
FMpre	25.0	-
FMeco II	-	25.8
Dried distillers grains	16.4	17.0
Wheat bran	10.0	10.3
Meat bone meal	8.38	8.65
Poultry fat	6.38	3.32
Sodium chloride	0.900	0.930
Choline chloride	0.088	0.088
Vitamin E	0.004	0.004
Minerals and vitamins premix ^1^	1.348	1.308

* FMpre: diet with feather meal premium; FMeco II: diet with feather meal economy II. ^1^ The premix used in the diets was a commercial preparation designed to meet the recommended requirements for vitamins and minerals. The detailed ingredient composition of the premix was not available.

**Table 3:** Chemical composition of feather meals (FMs) as an ingredient used in both trials.

Parameter	Unit	First Trial		Second Trial	
		FMpre *	FMeco I *	FMpre *	FMeco II *
Dry matter ^1^	g/kg	958	950	958	964
Crude ash	g/kg DM	24.6	13.7	24.6	13.7
Crude protein		907	953	907	907
Crude fat		76.1	52.8	76.1	80.5
Calcium		4.96	2.03	4.96	3.56
Magnesium		0.381	0.266	0.381	0.298
Phosphorus		3.77	1.82	3.77	2.46
Sodium		1.22	0.631	1.22	0.631
Potassium		1.53	0.838	1.53	0.987
Chloride		2.00	1.45	2.00	1.35
Sulfur		19.8	22.8	19.8	21.6
Copper	mg/kg DM	5.30	6.92	5.30	7.49
Zinc		151	117	151	116
Iron		173	126	173	263
Manganese		12.8	14.9	12.8	18.4
Selenium		0.361	0.814	0.361	0.550

* FMpre: diet with feather meal premium; FMeco I: diet with feather meal economy I; FMeco II: diet with feather meal economy II; ^1^ DM: dry matter.

**Table 4:** Amino acid contents and shares (g/kg dry matter (DM) and g/100 crude protein CP) of feather meals (FMs) in both trials.

Parameter	First Trial				Second Trial			
	FMpre *		FMeco I *		FMpre *		FMeco II *	
	DM ^1^	CP ^2^	DM	CP	DM	CP	DM	CP
Essential amino acids								
Arginine	61.9	6.82	66.1	6.94	61.9	6.82	62.1	6.85
Histidine	6.18	0.681	5.67	0.595	6.18	0.681	5.37	0.592
Isoleucine	43.4	4.79	45.5	4.77	43.4	4.79	43.4	4.79
Leucine	73.0	8.05	76.5	8.03	73.0	8.05	73.6	8.11
Lysine	20.8	2.29	18.9	1.98	20.8	2.29	17.9	1.97
Methionine	5.53	0.610	4.41	0.463	5.53	0.610	4.50	0.496
Phenylalanine	42.9	4.73	44.8	4.70	42.9	4.73	42.4	4.67
Threonine	40.3	4.44	41.2	4.32	40.3	4.44	38.9	4.29
Valine	65.1	7.18	70.5	7.40	65.1	7.18	43.3	4.77
Non-essential amino acids								
Alanine	44.5	4.91	45.5	4.77	44.5	4.91	43.3	4.77
Asparagine	61.1	6.74	63.4	6.65	61.1	6.74	60.7	6.69
Cysteine	45.0	4.96	53.8	6.65	45.0	4.96	44.1	4.86
Glutamine	98.6	10.9	101	10.6	98.6	10.9	97.2	10.7
Glycine	73.0	8.05	75.3	7.90	73.0	8.05	70.3	7.75
Proline	89.1	9.82	94.6	9.93	89.1	9.82	89.8	9.90
Serine	97.5	10.7	106	11.1	97.5	10.7	100	11.0
Tyrosine	25.8	2.84	27.4	2.88	25.8	2.84	24.9	2.75

* FMpre: diet with feather meal premium; FMeco I: diet with feather meal economy I; FMeco II: diet with feather meal economy II; ^1^ DM: dry matter; ^2^ CP: crude protein.

**Table 5:** Chemical composition of the diets including the feather meals (FMs) in both trials.

Parameter	Unit	First Trial		Second Trial	
		FMpre *	FMeco I *	FMpre *	FMeco II *
DM ^1^	g/kg	918	914	918	947
Crude ash	g/kg DM	75.7	66.5	75.7	74.7
Crude protein		373	383	373	401
Crude fat		120	124	120	90.3
Crude fiber		25.7	28.4	25.7	30.7
Nitrogen-free extract		329	315	329	355
Starch		312	354	312	339
Sugar		33.8	33.2	33.8	19.2
ME ^2^	MJ/100 g	1.54	1.55	1.54	1.53
Calcium	g/kg DM	15.5	14.0	15.5	14.2
Magnesium		1.39	1.50	1.39	1.51
Phosphorus		10.0	10.3	10.0	10.4
Sodium		5.49	4.63	5.49	5.59
Potassium		5.09	5.04	5.09	5.51
Chloride		8.93	7.69	8.93	9.24
Sulfur		7.63	7.92	7.63	7.85
Copper	mg/kg DM	15.6	20.7	15.6	17.6
Zinc		91.9	172	91.9	160
Iron		79.6	186	79.6	204
Manganese		7.74	41.7	7.74	53.0
Selenium		0.480	0.463	0.480	0.466

* FMpre: diet with feather meal premium; FMeco I: diet with feather meal economy I; FMeco II: diet with feather meal economy II; ^1^ DM: dry matter; ^2^ ME: metabolizable energy.

**Table 6:** Amino acid contents (g/kg dry matter (DM) and g/100 crude protein (CP)) of complete diets in both trials.

Parameter	First Trial				Second Trial			
	FMpre *		FMeco I *		FMpre *		FMeco II *	
	DM ^1^	CP ^2^	DM	CP	DM	CP	DM	CP
Essential amino acids								
Arginine	24.3	6.51	24.2	6.32	24.3	6.51	26.3	1.28
Histidine	4.82	1.29	4.78	1.25	4.82	1.29	5.67	1.41
Isoleucine	15.1	4.05	15.4	4.02	15.1	4.05	17.2	4.29
Leucine	28.7	7.69	29.0	7.57	28.7	7.69	30.4	7.58
Lysine	10.5	2.82	10.3	2.69	10.5	2.82	11.9	2.97
Methionine	4.64	1.24	4.34	1.13	4.64	1.24	4.51	1.12
Phenylalanine	17.2	4.61	17.3	4.52	17.2	4.61	18.5	4.61
Threonine	15.2	4.08	15.7	4.10	15.2	4.08	16.0	3.99
Valine	22.2	5.95	22.3	5.82	22.2	5.95	25.5	6.36
Non-essential amino acids								
Alanine	19.9	5.34	19.3	5.04	19.9	5.34	21.4	5.34
Asparagine	25.8	6.92	25.4	6.63	25.8	6.92	27.9	6.96
Cysteine	9.47	2.54	10.1	2.64	9.47	2.54	15.0	3.75
Glutamine	58.9	15.8	60.3	15.7	58.9	15.8	65.2	16.3
Glycine	31.5	8.45	29.8	7.78	31.5	8.45	33.3	8.30
Proline	36.0	9.65	33.4	8.72	36.0	9.65	40.5	10.1
Serine	33.6	9.01	34.1	8.90	33.6	9.01	35.1	8.75
Tyrosine	10.5	2.82	10.9	2.85	10.5	2.82	10.5	2.62

* FMpre: diet with feather meal premium. FMeco I: diet with feather meal economy I. FMeco II: diet with feather meal economy II. ^1^ DM: dry matter. ^2^ CP: crude protein.

**Table 7:** Apparent nutrient digestibility (%) of the complete diets with feather meals (FMs) fed to dogs in two trials (mean ± SD).

Item	First Trial		*p*-Value	Second Trial		*p*-Value
	Fmpre * (*n* = 6)	FMeco I * (*n* = 6)		Fmpre * (*n* = 6)	FMeco II * (*n* = 6)	
Organic matter	77.3 ± 1.64	76.3 ± 2.70	0.427	77.3 ± 0.948	75.6 ± 1.20	0.027
Crude protein	78.1 ± 2.54	78.2 ± 3.85	0.986	78.5 ± 2.01	78.1 ± 1.74	0.720
Crude fat	91.3 ± 1.02	90.7 ± 1.48	0.458	89.5 ± 4.16	86.4 ± 0.571	0.054
Nitrogen-free extract	76.6 ± 1.41	74.8 ± 2.25	0.122	76.5 ± 1.19	75.7 ± 1.51	0.305

* FMpre: diet with feather meal premium. FMeco I: diet with feather meal economy I. FMeco II: diet with feather meal economy II.

**Table 8:** Fecal characteristics of dogs fed complete diets with feather meals (FMs) in both trials (mean ± SD).

Item	First Trial		*p*-Value	Second Trial		*p*-Value
	FMpre * (*n* = 6)	FMeco I * (*n* = 6)		FMpre * (*n* = 6)	FMeco II * (*n* = 6)	
Fecal consistency score	3.23 ± 0.172	3.24 ± 0.478	0.748	3.19 ± 0.283	3.05 ± 0.141	0.457
Fecal form score	3.11 ± 0.114	3.09 ± 0.370	0.936	3.11 ± 0.251	3.03 ± 0.129	0.423
Fecal output (g DM ^1^/day)	35.3 ± 2.17	36.9 ± 5.52	0.536	39.8 ± 1.25	42.9 ± 1.68	0.004
DM (%)	29.4 ± 0.508	29.9 ± 2.14	0.609	30.2 ± 1.78	30.0 ± 2.02	0.864

* FMpre: feather meal premium; FMeco I: feather meal economy I; FMeco II: feather meal economy II; ^1^ DM: dry matter.

## Data Availability

The original contributions presented in the study are included in the Diss. of Shulten [37]; further inquiries can be directed to the corresponding author.
